# Nurses' perceptions and experiences of communication in the operating theatre: a focus group interview

**DOI:** 10.1186/1472-6955-5-1

**Published:** 2006-02-08

**Authors:** Debra Nestel, Jane Kidd

**Affiliations:** 1 Department of Biosurgery and Surgical Technology, Division of Surgery, Oncology, Reproductive biology and Anaesthesia, Imperial College London, c/o Clinical Skills Centre, 2^*nd *^floor, St Mary's Hospital, QEQM Wing, Praed Street, London, W2 1NY; 2 Reader in Communication Skills, WMS Division of Medical Education, University of Warwick, Coventry, CV4 7AL

## Abstract

**Abstract:**

Nurses' perceptions and experiences of communication in the operating theatre: a focus group interview

**Background:**

Communication programmes are well established in nurse education. The focus of programmes is most often on communicating with patients with less attention paid to inter-professional communication or skills essential for working in specialised settings. Although there are many anecdotal reports of communication within the operating theatre, there are few empirical studies. This paper explores communication behaviours for effective practice in the operating theatre as perceived by nurses and serves as a basis for developing training.

**Methods:**

A focus group interview was conducted with seven experienced theatre nurses from a large London teaching hospital. The interview explored their perceptions of the key as well as unique features of effective communication skills in the operating theatre. Data was transcribed and thematically analysed until agreement was achieved by the two authors.

**Results:**

There was largely consensus on the skills deemed necessary for effective practice including listening, clarity of speech and being polite. Significant influences on the nature of communication included conflict in role perception and organisational issues. Nurses were often expected to work outside of their role which either directly or indirectly created barriers for effective communication. Perceptions of a lack of collaborative team effort also influenced communication.

**Conclusion:**

Although fundamental communication skills were identified for effective practice in the operating theatre, there were significant barriers to their use because of confusion over clarity of roles (especially nurses' roles) and the implications for teamwork. Nurses were dissatisfied with several aspects of communication. Future studies should explore the breadth and depth of this dissatisfaction in other operating theatres, its impact on morale and importantly on patient safety. Interprofessional communication training for operating theatre staff based in part on the key issues identified in this study may help to create clarity in roles and focus attention on effective teamwork and promote clinical safety.

## Background

The importance of teaching and learning about communication with patients is well established within nursing [1]. Less emphasis is placed on learning about communicating with other health care professionals and especially communication within specialised working environments such as the operating theatre (OT).

Media reports in the United Kingdom have revealed poor outcomes for some patients as a direct consequence of ineffective communication in theatre or indirectly in conjunction with personal, technical and/or organisational issues. The Bristol Royal Infirmary Inquiry [2] investigating the high mortality rates of a paediatric surgical unit, identified that communications were "strained" and these exacerbated as a consequence of an anaesthetist who spoke out about what he observed in theatre. Many of the recommendations from the Inquiry were based on improving communication both within and outside of the OT promoting basic qualities of respect, honesty and openness between the health professionals, National Health Service management, patients and the public.

There are many journal publications that provide guidance to nurses for communication in the OT [3-20]. The content and number suggest that problems of communication in theatre have long been recognised. However, there is much less empirical research to support what is apparently well known. General themes are identified in this anecdotal and advisory literature targeting nurses. Reference is made to the importance of verbal and non-verbal communication in the OT with emphasis placed on active listening. There is little recognition of the unique environment in which the communication takes place or factors external to the setting that may influence the way in which participants interact. This literature also deals with stereotypes such as the overtly combative communication style of surgeons or with communication crises rather than everyday occurrences.

Importantly, the research base of communication within the OT is increasing. Two papers published by a research team in Canada are based on observational and interview data and analysed using a grounded theory approach. In the first study, Lingard et al [21] report the following key findings:

### • Patterns of communication

Observed communicative events in the OT were thematically categorised into discussions about time (patient cancellations, sending for the next patient; preparation of the theatre); resources (booking and provision of equipment; personnel); roles (responsibilities, constraints) and relationships; safety and sterility (aseptic technique) and situation control (temperature regulation, data recording activities). Although communications were complex, identifiable patterns also emerged such as the rhetoric demonstrated by surgeons when making requests of nurses. Rather than issuing a command, surgeons asked a question or made a statement to achieve their goal.

### • Sites of tension

Communicative tension occurred regularly in relation to themes outlined above and these tensions often had a ripple effect, spreading beyond those involved in the initial exchange to other members of the OT as well as beyond the original context of the tension.

### • Impact on novices

Communication in the OT influences the socialisation of novices with evidence of mimicry of senior staff or withdrawal from the communication sphere under certain circumstances. Novices also demonstrated different communication styles depending on who was present in the OT seeming able to communicate effectively with either the surgical or nursing teams but not necessarily both simultaneously.

The second study developed the notion of communication as a critical component in the formation of professional identities [22]. Shared "talk" facilitates role acquisition and maintenance enabling novices to find an identity with members of their professional group. Based on their observations and interactions, novices also construct roles of others within the OT. The results of the focus group study revealed that although nurses, surgeons and anaesthetists largely had shared understanding of the technical aspects of their roles, there was dissonance in relation to the non-technical or "professional/relational" issues of authority, motivation and value. Nurses and surgeons were divided in attributing motives for adopting particular communication styles. That is, the same comment can be interpreted differently according to whether the speaker was perceived to be assuming a patient advocacy role or desiring power. These studies are set within a Canadian context and may not be appropriate to other settings and countries.

Communication in the OT has been documented in studies exploring the role of the OT nurse. These studies have employed a combination of methodologies including observations, interviews and document analysis [23,24]. Tanner and Timmons [25] use Goffman's notion of "backstage" and "frontstage" to describe behaviours of medical and nursing professionals in different settings with the OT approximating "backstage." They found evidence that doctors and nurses perceived the OT as a "private environment" in which the nature and content of conversations differed to those in other settings and especially those in which patients were present (and conscious). Although interpersonal professional relationships appeared relaxed and informal "backstage", the authors suggest that this should not be mistaken for the absence of hierarchy.

At the request of senior members of the surgical department at a London teaching hospital, the authors were invited to develop an educational intervention to support the acquisition and maintenance of communication skills of trainee surgeons. Given the multi-professional nature of the OT, focus group interviews were conducted with different professional groups exploring their perceptions and experiences of communication in theatre. This information, together with observations of interactions in the OT will inform the development of a communication programme. The aim of this paper is to report nurses' perceptions and experiences of communication in the OT that were identified in the focus group interviews as part of this broader project. This provides an evidence base for the development of interventions that may enhance teamwork with benefits for all OT staff.

## Methods

After organisational ethics approval had been obtained, OT nurses were invited by a doctor from a department of surgery to take part in a focus group interview. The sampling was both purposive and convenient accommodating participants' rosters as well as nurses from general and specialist theatres. The interview was scheduled for one-hour and was audio-recorded after obtaining consent from participants.

**Figure 1:**
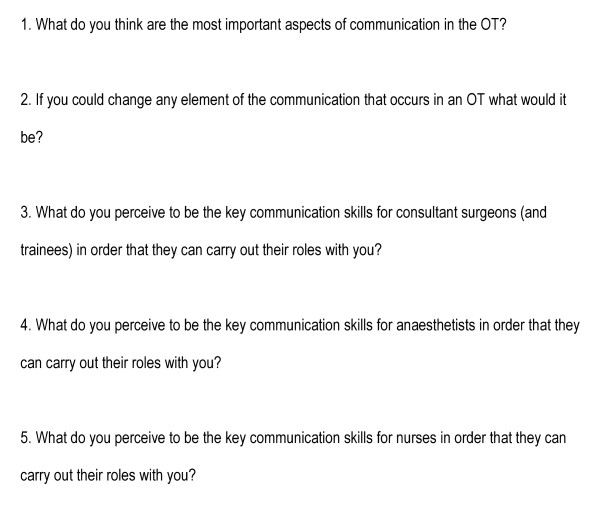
Topic guide for interviews with operating theatre nurses.

Prior to commencing the interview, nurses completed a brief questionnaire. The concept of focus groups as a means of eliciting information was outlined and participants were encouraged to build on each other's ideas as they were expressed. The topic guide was introduced so that participants were aware of the range of topics that would be explored (Figure 1). Anonymity was assured. All participants verbally consented to participate and were sent a summary of conclusions. Interviews were conducted by one researcher (DN). A transcript of the interview was made (JK) and emergent themes identified and negotiated between researchers (DN & JK)

## Findings

Seven female nurses participated in the interview. Five were white Caucasian, two were Asian and they ranged in age from 31 to 50 years. Five reported that they had completed communication training as part of their professional development and that this included assertiveness training and communication for management.

All nurses actively participated and were at times highly animated venting what were obvious frustrations at personal and professional levels. The interview lasted 90 minutes and could easily have continued but was brought to a close to stay within reasonable limits of what had been intended. Question 5 was not given as much attention as questions 1–4 due to the pressure of time and that there was overlap between questions. The following results summarise the responses. Themes are listed and then illustrated with verbatim quotations in italics from the interview. Paragraph quotations from individual nurses are coded to demonstrate the distribution of responses.

### 1. What do you think are the most important aspects of communication in the OT?

Nurses were unanimous in identifying the importance of listening for effective communication. One participant described the need to *"listen hard" *highlighting the acuity with which this skill was practised. Two-way understanding was also considered important. That is, checking that individuals understand what has been asked of them as well as confirming that they have been understood. Speaking clearly *("Avoid mumbling") *and using courteous language *("No abusive language") *were cited. Paralinguistic cues such as tone of voice was valued. Delivering messages to the right person and finding out their name and role was also considered important.

*... when you are communicating information to people I think they need to identify who they are actually giving the information to because a lot of the time, you know, they will say I told somebody but they don't, they can't really identify who that somebody is. *Participant 4

Nurses identified written communication as important. A "communication book" was used to convey a range of critical information for the OT – changes to schedules, equipment being serviced, staff on sick leave and personal messages.

### 2. If you could change any element of the communication that occurs in an OT what would it be?

There was an overwhelming response that respect, common courtesies and manners were essential and often absent.

*That it doesn't matter what level you are, what hierarchy, whether you are a sister or not, you speak to everybody civilly. *Participant 2

One nurse stated that the decisive non-verbal communication act *"throwing instruments on to the floor" *should stop. Most nurses nodded in agreement and when asked how often this occurred, there was agreement that this happened up to four times a year.

Organisational issues impacted communication. Although nurses acknowledged the importance of induction programmes, they strongly urged that the programme for medical staff be reviewed so that the frequent turnover of juniors (every 3 to 6 months) would not take up their time. Nurses were adamant that they did not want medical induction to form part of their role.

*... you know we have to go through the rigmarole... you get juniors (trainee surgeons) come up and say I don't know how to use this, I wasn't shown how to, I don't know what to do. *Participant 1

*...we already take on a lot of their roles and some things that we don't know about it's only their colleagues who know how to do it and they need to speak to their colleagues and it's their colleagues who need to train them how to do it and not us because we do not know ourselves. It's like booking patients on the computer that you know they have to teach themselves or teach each other how to do that. *Participant 3

### 3. What do you perceive to be the key communication skills for surgeons (and trainees) to carry out their roles?

In response to the questions about communication skills for different professional groups, common themes emerged as well as repetition from earlier questions. Nurses were especially vocal and energetic in responding to this question. Common courtesies and respectful behaviours were identified as key. These were illustrated with examples that questioned professional competence, over running schedules, starting late and sending for patients.

I have, I have surgeons that turn round to me and say that I have never seen this nurse before. I do not know what she can do. I don't want her. Participant 5

*Certainly there should be a discussion so that if you are going to overrun...they say, send – and it's like quarter to four or something, like that you know you're going to overrun and it's just assumed that you are going to stay and it's just nice common courtesy to actually ask the staff is everyone is willing to stay. *Participant 1

*Sometimes, operations don't go to time, you can never time an operation. Therefore it's going to overrun. Therefore, you know the patient is not going to get done. I mean it's happened this week and the patient, to my knowledge, has still not been operated on because he can't get allocated time in that theatre. *Participant 7

*It's like when you're trying to start a list in the morning you have the patient there, you have the anaesthetist there, you have the nurses there, they're (surgeons) doing a ward round. There is no common courtesy to ring to say they are going to be late. You are waiting to start so therefore you are delayed in the morning. Therefore, it's going to be a knock on effect in the afternoon. *Participant 2

Nurses reported inadequate communication between surgeons. Nurses were often expected to act as a *"go between." *There was frustration with the experience that surgeons could be courteous to one another but not to nurses even within the same communicative event.

*So what we are saying is that consultants don't communicate well with each other. They have some sort of etiquette going on whereby the language that they use towards each other is totally different... I'll give you a prime example is that I was running a list in which we were using the x-ray. Another consultant came, I walked out to the door. He shouted at me about why we were using the equipment at that time of the day. I pushed the door open and said "Don't tell me, tell the surgeon". The way that he spoke to him was totally different and it was almost as though "it's okay" right I don't mind you using it and we need to come to some sort of arrangement but he is shouting at me as if it his right to use the equipment now. So the way that they interact with each other is totally different. They are not honest with each other. They will slate each other behind their backs but they will not say anything to their face, never. *Participant 7

Nurses thought it important that accurate (e.g. the names of instruments) and complete information be provided.

*When we when we are given specimens you say they might say "specimen" you might say "for histology" they might want it dry, frozen sections they might not always tell you in formalin sometime you have to keep prodding for bacteriology, cytology all these, why can't they say the appendix for histology in formalin or whatever. Yes we know that some junior nurses might not know not always those sorts of things as well they assume you know and mistakes can happen. Specific instructions so it's a two way thing they're saying now that they want us to acknowledge their commands but they're not acknowledging ours as well so it's a two way game. It's a team. *Participant 1

Discussion extended to several related topics that are likely to influence communication and included notions of effective teams.

*I truly believe that we are working our damndest to work as a team. Doctors are still, and this is consultant all the way down, are still working to their own agenda and they do not believe that they are part of our team and they are part of our team but they don't believe. I am sure they don't believe that they are, they're a stand alone team and we're a team here and they're a team there and they'll pick up what they need but we can't take anything from them. Does that make any sense? *Participant 4

This theme was elaborated in discussions about roles and responsibilities of members of the OT with emphasis on perceptions of the role of an OT nurse. Many of these views were expressed with intense frustration and illustrated with specific examples (e.g. draping patients, cleaning the theatre, answering mobile telephones).

... expect us to be secretaries in the theatre as well as doing the work ...

Participant 3

*We know what our professional role is, we know what our professional role is but they don't. *Participant 7

*I'm sorry to say that it is the surgeon's responsibility to make sure that a patient is positioned the way you want it and the way it's been draped. It is the operating surgeon's responsibility. It is actually not a nurse responsibility because a nurse can only provide you with the equipment and the necessary tools for you to perform the surgery and assist you but she is actually not there to know how you are going to approach the operative procedure. *Participant 5

*I tell you what they are talking about the waiting time is what they don't understand is when they've walked out of the operating theatre the nurses still have to clean the floor of all the operating theatres, empty the bags, you know these things take time, it doesn't happen its' not a miracle you know we are supposed to clean the tables and the trolleys and they may see it as a natural break. *Participant 2

*There are other factors like mobile 'phones. You are probably the only person in the theatre...bleeps going maybe... You are expected to be hands here, there and everywhere. Like, can you answer my mobile phone? And while you are, you know concentrating on that, that's when he wants the diathermy. *Participant 4

Nurses distinguished themselves from surgeons in relation to patient advocacy. This was illustrated in examples of sending for patients and leaning on patients.

*You see I think I think we look at the patient, we're the patient's advocate. When they are leaning all over the patient, they don't care and if you tell them please that is a body under there that is, I mean how would you like it if that was your wife you know you should not lean on the patient. I had a surgeon and when they had finished leaning on the patient the towel clip was actually imprinted on that patient's skin. *Participant 1

Nurses also identified strongly expressed emotions.

*The only problem is if they don't tell us in advance and it is something we haven't got in the department. I mean it's beyond our control we can't give it to them. Again if we have it in the department and it's not clean and they have to wait for it to be sterilised so it might compromise time again and they might get angry as well you know being impatient you know, shouting "When is it going to be ready?" "How long is it going to take? *Participant 7

Nurses expressed some frustration with their constant adaptation to circumstances beyond their control in relation to taking breaks (or not).

*Can I just say, can I just say the majority of the people in this room will turn to you and say that half of us never get a proper break during the day because we'd rather do the operating and try and finish the list. *Participant 5

Power, hierarchy and acknowledgement were sources of frustration for nurses.

*We've moved away from Yes sir, we've moved away from that a long time ago. We do anticipate, we do give them what they need. We don't always get acknowledgement from, from our point of view and if we felt that we needed to say something to them i.e. yes that's done then we will tell them that but if we don't we won't. *Participant 2

A sense of helplessness was expressed in relation to training opportunities.

*I think it's to be a bit more patient and compassionate especially when we are trying to train nurses up to be as competent as they want them to be and you know it is to give them that opportunity to develop that role that they're put in there to do. Not sort of just brush them aside and say you know, I'm too busy you know I don't want this because you know, I mean it is a teaching hospital and we are supposed to teach people and to train them and that opportunity is not given with compassion then it's you know, its never ever going to work. *Participant 6

Nurses were adamant in their views that trainees should not follow the examples of consultant surgeons. The nurses also identified *"a bit of a barrier if they do not speak English as their first language." *Nurses also suggested that consultants do not communicate well with trainee surgeons and this has implications for nurses' roles.

### 4. What do you perceive to be the key communication skills for anaesthetists to carry out their roles?

Unlike the response to questions about surgeons, anaesthetists did not generate as much discussion nor was the response as energetic. They were described as *"more approachable" *than their surgeon colleagues. Nurses reported that anaesthetists sometimes seemed isolated from the rest of the OT team.

*They don't appear to have much communication with the surgical team. It is with the anaesthetic person that's there that they communicate with... *Participant 1

Again, the *"go-between" *role expected of nurses was outside of their own role perception.

*To make it work, yeah, is for the anaesthetist to communicate with the surgeons that they are working with and not going through the nurse to do the communication for them. That is the key issue. The key issue with a lot of anaesthetists is that is when they are not happy to perform a particular surgery they will not go and communicate with the surgeon and say "I am not happy in doing it." They want you to tell them that YOU, you personally is not happy. *Participant 6

Like the surgeons, the anaesthetists were also criticised for starting late and for not keeping nurses informed.

*They're supposed to start the list, I mean some of them actually do phone and say they're going to be late and that's fair enough. That's appreciated and that's anybody but some of them, they don't care as much as you've spoken to them and said look the list is supposed to start at a certain time everybody's here and why aren't you? You know. *Participant 2

### 5. What do you perceive to be the key communication skills for nurses to carry out their roles?

Responses initially focused on written rather than verbal communication and then moved to administrative issues before describing interpersonal communication. Written communication in the form of hard copy documentation and memos of policy and changes in practice.

*Documentation because there are so many of us, there are so many of us it is difficult to talk to everyone, so it's documentation, it's getting a memo out, getting something in the communication book so that or putting information in the appropriate place so that everybody gets it or giving information to the key people who can cascade it down. *Participant 4

Electronic communication was regularly used to exchange information but there are problems with the system and limited access. During long cases it was thought appropriate to read email but not all theatres have this facility.

Meetings that are uni-professional were thought valuable but were reported as often lacking in structure and there were difficulties finding dedicated "protected" time.

*We had one for the first time, a structured one, on a particular subject and what came out of it was very very good because we didn't deter from that. Did you feel that? That we didn't deter from the subject so that's something that nurses aren't very good at – is using the forum for what it's been for what it's supposed to be used for or what normally happens is that you have your agenda then you go off on a tangent... *Participant 3

There was a desire for inter-professional meetings although the content and format were not explored.

Unlike responses to other questions nurses referred to the role of nonverbal communication.

*I think if you've got a good rapport with your runner (Circulating nurse), you can, you know use nonverbal communication. *Participant 2

*Definitely, yeah you can.... Definitely pick up what you want and if you have an excellent runner or an experienced runner we don't have to say anything. It's there or it's waiting. *Participant 4

Although the need for training was recognised as crucial, nurses' experiences of surgeons did not always support such professional development.

But they don't give us the chance to teach our juniors. They are allowed to bring junior doctors there and teach them and train them and what have you and they're allowed to do that during emergency surgery and whatever at any time and they don't allow us to take a nurse to double scrub with someone to teach them or to actually let you know, they want it done, now, now, now, now (finger clicking at same time) they cannot wait.

Participant 6

## Discussion

The results suggest that communication in the OT is diverse and complex. The key behaviours for effective communication included a range of verbal and non-verbal skills. Active listening was very important as well as basic interpersonal skills such as clarity of speech, being polite and courteous. The most notable themes appeared to be factors that indirectly influence communication, especially confused and conflicting role perceptions. Responses to questions in this interview focused on nurses' roles but it is possible that perceptions of surgeons' and anaesthetists' roles also lacked clarity. In contrast to the findings of Lingard et al, [26,27] the nurses in this study strongly suggested that there was confusion in relation to the perception of technical aspects of roles (as well as non-technical aspects). However, the content of nurses' responses fell within the four categories of patterns of communication described by Lingard et al [28] and that these were also sites of tension suggesting the relevance of these issues across national boundaries.

Nurses in this study appeared to be immensely frustrated by the expectations others' have of their roles. Much of this frustration stemmed from working outside of their role and compensating for what they considered were deficiencies in other team members. The role of the OT nurse has proven difficult to define and there is pressure from within the profession to do so [29-31]. Although general nurse role definitions include patient-centred and health-wellness models of care, at least practically, nurse roles in the OT are strongly influenced by technical and task-oriented aspects of care [32-36] and that the medical profession exerts considerable influence over OT nurse work [37]. However, the nurses in this study seemed confident about their own role boundaries. In their view it was others who neither appreciated nor respected these boundaries. This contrasts with the OT nurses interviewed by McGarvey et al [38] who had difficulty defining their role although there was agreement that it was "complex and specialised." Several authors have argued that nurse-doctor communication in the OT is strongly hierarchical but that it is also increasingly negotiated reflecting broader societal trends [39-42]. There appeared to be few opportunities for the nurses in this study to negotiate relationships but they were eager to do so.

This was a study on communication, not roles although the latter significantly influences the quality of communication and was foremost in these nurses' minds. There was certainly room for improvement in communication in the OT and that the current mode of communicating was perceived as a maladaptive strategy. Nurses reported little sense of satisfaction with communication and other aspects of their role.

It was apparent that the whole context of work and not just specific skills were important for improving communication. This sits neatly within the role performance framework for OT nurses developed by McGarvey et al [43] in which the key dimensions of OT nurse work were context (e.g. atmosphere, technology, physical aspects etc), role performance (e.g. socialisation into the role, accountability etc) and personal and professional characteristics of nurses. Each dimension influenced the other and that they were all important.

Nurses' comments in this study reinforced professional stereotypes of surgeons and nurses in the OT supporting the anecdotal literature [44]. The consensus of views suggests that these stereotypes were valid. However, it would be helpful to have actively explored during the interview nurses' experiences outside of the stereotypic. Timmons and Tanner [45] describe "emotional labour" and the "hostess role" of nurses in the OT that accurately reflected the actual roles that nurses in this study described for themselves. That is, these nurses felt that the enactment of their roles was in someway looking after surgeons and attending to their needs although this conflicted with the ideal image of themselves as equal partners in a professional relationship.

Nurse-patient communication was rarely mentioned in this interview except that nurses believed they were patient advocates. McGarvey et al [46] identified several barriers to nurse-patient communication in the OT including nurses wearing masks, the absence of name badges, that conscious patients arrived in theatre laying down and without hearing aids, glasses or false teeth. Even though observers identified opportunities to minimise these barriers, nurses chose not to do so suggesting that nurses were interacting (or not) with patients (by exerting their power) in the same way that the nurses in this study reported they were subordinated to surgeons (exerting their power) through communication.

The findings suggest that these nurses would be receptive to inter-professional training targeted at improving communication. This is most likely to be successful by tackling the broader issues of teamwork in which communication sits. Using high fidelity simulation to recreate communication scenarios in the OT may be valuable in prompting overt discussion and reflection not only of skills but the important issues of attitudes, role clarity and effective functioning within teams.

Nurses' responses were made in the context of a strong hierarchical structure reflecting those in other high intensity professions such as aviation where there are established communication protocols. Anaesthetists have pioneered the application of training principles from aviation in crisis resource management (CRM) using high fidelity simulations [47-49]. Although the overt goals of CRM are effective use of resources in anticipating and responding to adverse events, the skills required are rooted in effective communication, perception, decision-making and leadership.

Management literature may also provide valuable insight into communication and effective functioning of the OT. Nurses' responses to the questions in this study suggest management styles and team functioning that are ineffective. The works of Belbin [50], Handy [51] and Kanter [52] offer different perspectives on management, leadership and team functioning making explicit individual and systems factors that influence "productivity."

Perhaps what is crucial is that unsatisfactory work practices are identified and remedied because of their influence on novices. Lingard et al [53,54] describe the impact on trainees within the OT while Lave and Wenger [55] "communities of practice" more broadly describe the importance of work practice on the "professionalisation" of trainees. Although it is difficult to assess the impact on staff and patients of ineffective communication there is evidence that outcomes are compromised in some circumstances [56].

Focus groups are an effective means of developing ideas expressed by individuals. There were very few disagreements between participants. Focus groups may be influenced by the strong views of a couple of members. However, in this interview all participants contributed and there was no disagreement in statements, only in the voracity of expression. The numbers of participants were small and only one interview was conducted. It would be valuable to explore the views of nurses with less experience and working in smaller hospitals. Observational studies would provide further insight. Shortcomings of the study include the small sample and that the views of nurses working in a large teaching hospital may not reflect all OT communications.

## Conclusion

This paper provides evidence from the perspectives of OT nurses of the communication behaviours within the OT in a United Kingdom setting. Key skills deemed essential include listening, courteous behaviour, acknowledging requests and clear accurate speech. Communication in the OT is compromised in relation to the absence of basic interpersonal skills and appreciation and respect for different professional roles. Current practice seems to be based on making assumptions about the knowledge and skills of other professional groups. There appeared to be little or no opportunity to check and clarify assumptions resulting in an unsatisfactory state of communication.

While there is some evidence that there is an impact on patient health, if the communication in this environment can be enhanced it may improve job satisfaction and create a positive environment in which trainees can learn appropriate behaviours and attitudes. The study suggests that there is scope to enhance the communication skills of all OT professionals and that focused interprofessional training may be valuable.

## Pre-publication history

The pre-publication history for this paper can be accessed here:


